# Engineered ice-binding protein (*Ff*IBP) shows increased stability and resistance to thermal and chemical denaturation compared to the wildtype

**DOI:** 10.1038/s41598-024-53864-w

**Published:** 2024-02-08

**Authors:** Yewon Nam, Dieu Linh Nguyen, Trang Hoang, Bogeun Kim, Jun Hyuck Lee, Hackwon Do

**Affiliations:** 1 Research Unit of Cryogenic Novel Material, Korea Polar Research Institute, Incheon, 21990 Republic of Korea; 2 Division of Life Sciences, Korea Polar Research Institute, Incheon, 21990 Republic of Korea; 3 Department of Polar Sciences, University of Science and Technology, Incheon, 21990 Republic of Korea

**Keywords:** Ice-binding protein, Antifreeze protein, Cryopreservation, Chemical stability, Cold denaturation, DUF3494, Ice recrystallization inhibition, Thermal hysteresis, Biological techniques, Biotechnology, Molecular biology

## Abstract

Many polar organisms produce antifreeze proteins (AFPs) and ice-binding proteins (IBPs) to protect themselves from ice formation. As IBPs protect cells and organisms, the potential of IBPs as natural or biological cryoprotective agents (CPAs) for the cryopreservation of animal cells, such as oocytes and sperm, has been explored to increase the recovery rate after freezing–thawing. However, only a few IBPs have shown success in cryopreservation, possibly because of the presence of protein denaturants, such as dimethyl sulfoxide, alcohols, or ethylene glycol, in freezing buffer conditions, rendering the IBPs inactive. Therefore, we investigated the thermal and chemical stability of *Ff*IBP isolated from Antarctic bacteria to assess its suitability as a protein-based impermeable cryoprotectant. A molecular dynamics (MD) simulation identified and generated stability-enhanced mutants (*Ff*IBP_CC1). The results indicated that *Ff*IBP_CC1 displayed enhanced resistance to denaturation at elevated temperatures and chemical concentrations, compared to wildtype *Ff*IBP, and was functional in known CPAs while retaining ice-binding properties. Given that *Ff*IBP shares an overall structure similar to DUF3494 IBPs, which are recognized as the most widespread IBP family, these findings provide important structural information on thermal and chemical stability, which could potentially be applied to other DUF3494 IBPs for future protein engineering.

## Introduction

Cryopreservation is an important technique in modern biotechnology and has many applications in areas such as in vitro fertilization (IVF), stem cell research, and tissue engineering. Cryopreservation allows biological samples to be stored for long periods of time without compromising their viability, which is critical for research and clinical applications. Many organisms living in cold areas produce ice-binding proteins (IBPs) or antifreeze proteins (AFPs) as a defense mechanism against the damage caused by ice formation at subzero temperatures. (Although the term AFPs is more specific in explaining proteins with thermal hysteresis (TH) activity and/or ice recrystallization inhibition (IRI) activity^1,2^, the term IBPs has a broader conceptual scope, accurately representing the function of the proteins and encompassing the previously identified IBPs and AFPs. Therefore, in the present study, we used both AFP and IBP interchangeably to encompass the previously identified IBPs and AFPs^3^.) Glycosylated AFPs (AFGPs) and AFPs were first discovered in the blood serum of polar fish, such as the Antarctic notothenioid^4^, winter flounder^5,6^, and ocean pout^7^. AFPs have also been identified in insects, plants, and bacteria and categorized into different types based on their structural features^8^.

AFPs and IBPs can inhibit ice growth by binding to specific planes of ice and lowering the freezing temperature through a mechanism called absorption-inhibition^9,10^. Their binding properties are measured via the gap between the melting and freezing temperatures, referred to as thermal hysteresis (TH) activity. Previous research has primarily focused on the interactions between ice and IBPs (or AFPs) and the effects of their binding, such as lowering the freezing point and inhibiting the growth of ice crystals, which are unique abilities of biological molecules that are considered crucial characteristics for protecting host cells. Given that AFPs and IBPs can protect host cells from freezing, research has explored their potential applicability in preserving important mammalian cells such as gametes and embryos^11^. For instance, AFPIII, one of the most well-studied AFPs, has been reported to improve the preservation of sperm, oocytes, and embryos from various organisms and can be used with various freezing methods, including vitrification, chilling, and cryopreservation. Glycosylated AFGPs have also been investigated for the cryopreservation of sperm, oocytes, and embryos.

Dimethyl sulfoxide (DMSO), glycerol, ethylene glycol, methanol, and 1,2-propanediol are common membrane-permeable cryoprotective agents (CPAs) utilized in the cryopreservation or vitrification of mammalian cells and tissues, known as membrane-permeable cryoprotectants^11–13^. Among these, DMSO and glycerol are well-recognized CPAs primarily owing to their ability to reduce the formation of intracellular ice during cell freezing. Therefore, 10% DMSO or 20% glycerol is commonly utilized in cryopreservation procedures to maximize cell recovery after the freeze–thaw process, even in studies evaluating the effectiveness of AFPs or IBPs as CPAs^14^. Despite the discovery and study of various AFPs and IBPs that protect organisms from freezing, little is known about their chemical tolerance to CPAs. Therefore, understanding how these denaturants affect the stability and functionality of proteins under cryoprotective conditions is crucial for the development of improved cryopreservation methods and the design of proteins with enhanced stability in such environments.

The *Ff*IBP from *Flavobacterium frigoris* PS1 exhibits high TH activity and relatively strong recrystallization inhibition activity^15^. The *Ff*IBP structure is composed of parallel β-strands in a helical pattern, which is mainly stabilized by inner hydrophobic interactions and intramolecular hydrogen bonds with long α4^15^. Additionally, the *Ff*IBP has a capping head region that consists of two small turns and β-strands, which contain a disulfide bond. This disulfide bond increases the overall rigidity of the protein and reduces residual movement at the ice-binding site, thereby augmenting TH activity and protein stability^16^. These findings suggest that the introduction of additional disulfide bonds may further enhance TH activity and protein stability. Consequently, the present study aimed to investigate the stability and chemical resistance of *Ff*IBP in the context of CPAs and to establish functionally improved IBPs using molecular dynamics simulations.

## Results

### FfIBP mutagenesis guided by circular dichroism and molecular dynamics

Most hyperactive IBPs and AFPs contain more than one disulfide bond, which is one of the factors contributing to protein stability and TH activity^17^. Our previous study demonstrated that the structural rigidity of *Ff*IBP is positively correlated with its thermal stability and TH activity^15,16^. Based on these findings, we aimed to enhance the TH activity and thermal stability of IBP by introducing an additional disulfide bond in the structurally unstable regions in addition to the pre-existing disulfide bond.

**Figure 1:**
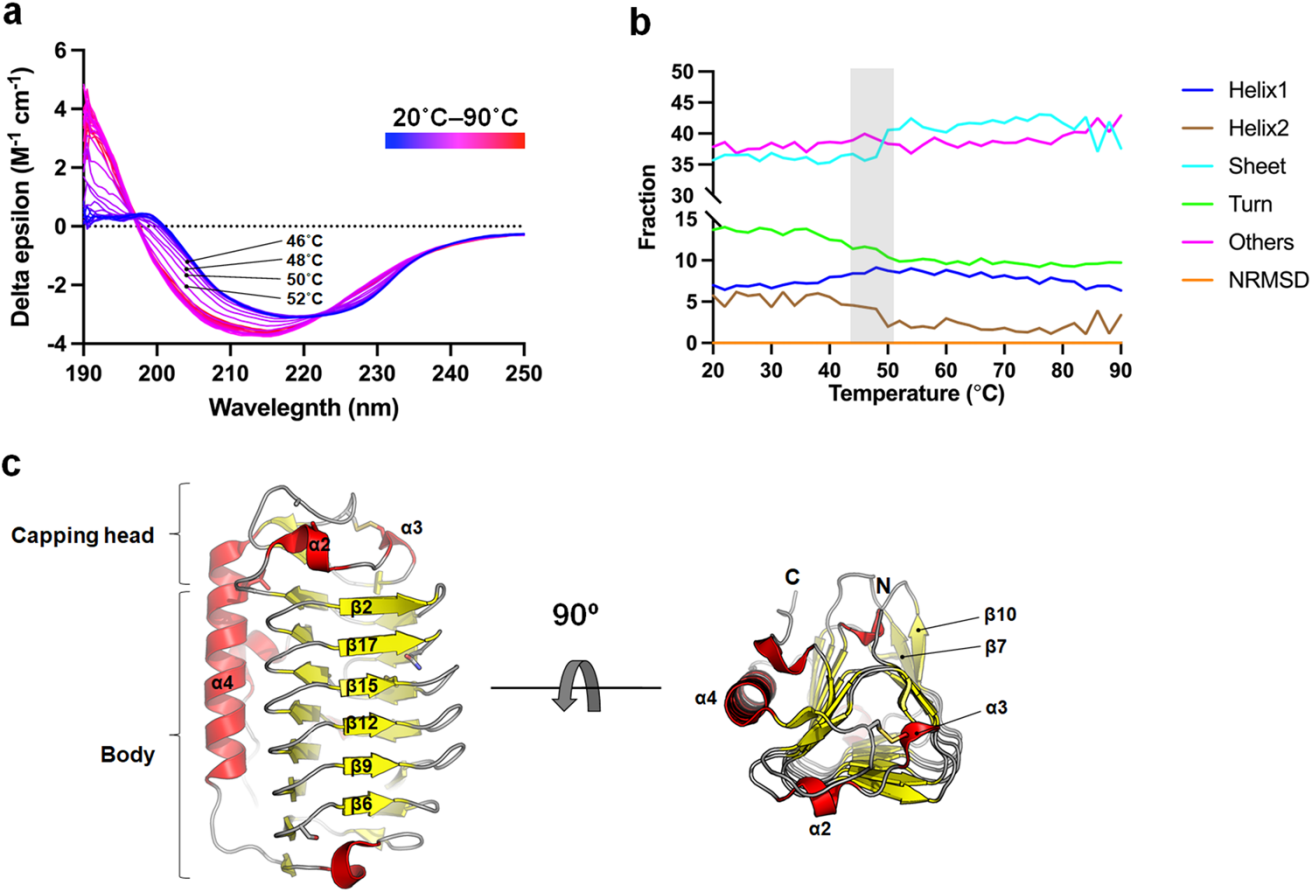
Circular dichroism (CD) spectroscopy of *Ff*IBP during the unfolding process and *Ff*IBP structure. (**a**) Temperature-dependent CD spectra of *Ff*IBP. Spectra were recorded in 10 mM Tris–HCl (pH 8.5) buffer from 190 to 250 nm with 0.5 nm steps and 0.5 s/step at a 1 mm path length. The spectra subtracted by baseline measured at each condition were averaged. The temperature was set from 20 to 90 ° with a 2 °C increment per step and the spectrum at each temperature is indicated with gradient color. (**b**) Secondary structure contents as a function of temperature. Amino acid propensities for helix, β-strand, turn, and other (mostly coil) structures at different temperatures were calculated using BeStSel^39^ and indicated with different color codes. (**c**) Overall structure of *Ff*IBP is visualized both in side and top views. sα-helix, β-strand, and turn structures are indicated with red, yellow, and grey colors, respectively. N- and C-termini are indicated by N- and C-, respectively.

To determine the ideal locations for introducing disulfide bonds in*to Ff*IBP, circular dichroism (CD) analysis was conducted to monitor changes in the secondary structure of the protein at various temperatures. Evaluating the propensity of a secondary structure during the unfolding process provides insight into the early steps of thermal unfolding^18^. The temperature range used was 20–90 °C, with a 2 °C increment per step. The results indicated that the structure of *Ff*IBP remained stable between 20 and 44 °C but began to unfold at 46 °C (Fig. 1a). The prediction of secondary structures at these temperatures indicated that the amino acid propensities of *Ff*IBP for the β-sheet structure slightly increased. However, compared with that at 20 °C, the ends of α-helices (Helix2) decreased from 5.7% to 1.9% (50 °C), and the turn structure also decreased in concert with increasing temperatures (Fig. 1b). This result indicates that backbone disorder is initiated in the helical region of *Ff*IBP by heat and that the helical and capping head regions of *Ff*IBP may be promising for introducing disulfide bonds and maximizing protein stability (Fig. 1c)^19^.

**Figure 2:**
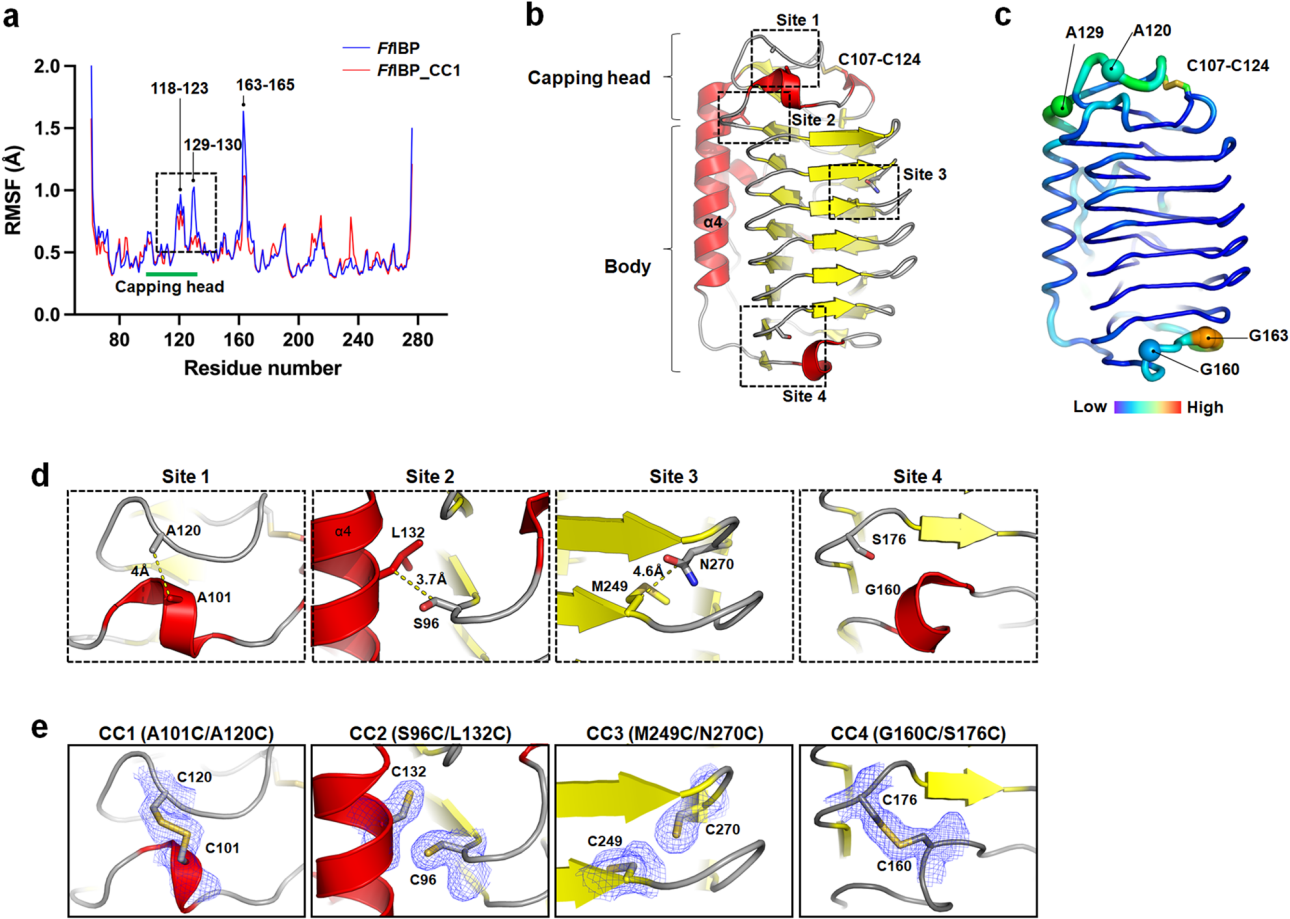
MD simulation and generation of *Ff*IBP mutants for disulfide bonds. (**a**) RMSF value for each residue during MD simulations. Flexible residues are indicated with residue numbers. (**b**) The overall structure of *Ff*IBP is depicted with a cartoon representation and mutation sites for the disulfide bonds are indicated with dotted squares. (**c**) The putty representation of the *Ff*IBP structure is colored by the RMSF value from the MD simulation, viewed from the same direction as in (**a**). Flexible residues are highlighted in different color codes. The value range is between 0.305 and 2.139 Å. (**d**) Close-up view of the residues mutated to cysteines for the disulfide bond. (**e**) The structures of mutants were determined via X-ray crystallography and the identical region shown in (**d**) is presented. 2Fo–Fc electron density maps contoured at 1.0 σ are displayed around the cysteines with blue mesh. MD, molecular dynamics; *Ff*IBP, engineered ice-binding protein; RMSF, root-mean-square-fluctuation.

Molecular dynamics (MD) simulations were performed at 200 nm to gain insights into the local conformational fluctuations of *Ff*IBP. The results demonstrated that the capping head region (Ser116–Ala136) and the bottom region (Gly160–Lys165) were the most flexible areas, whereas the residues in the body region were relatively stable, with an average root-mean-square-fluctuation (RMSF) value of 0.42 Å (Fig. 2a). The top loop of the capping head region (Asp118–Ala123) exhibited an average RMSF value of 0.85 Å. Another flexible region close to the capping head region is the edge of long helix4 (Asp128–Leu132). Specifically, the root mean square deviation (RMSD) of Ser130 increased by 1.0 Å. The bottom of the body region (Gly160–Lys165) also displayed a high degree of fluctuation starting from the short-turn region (Fig. 2b, c).

Based on the CD and MD simulation data, four mutants annotated as *Ff*IBP_CC1 (A101C-A120C), *Ff*IBP_CC2 (S96C-L132C), *Ff*IBP_CC3 (M249C-N270C), and *Ff*IBP_CC4 (G160C-S176C), possibly forming disulfide bonds, were generated (Fig. 2d). The criteria for choosing residues to mutate was i) the high degree of local fluctuation of the residues, ii) the Cβ distance between two residues, which should be close to 4 Å for disulfide bond formation, and iii) not close to the ice-binding site. The mutation site for *Ff*IBP_CC1 was located in the top loop of the capping head region, which contained a largely flexible residue Gly121. The *Ff*IBP_CC2 is located at the interface between the body domain and the long α-helix (α4). The thermal stability analysis suggests that the α-helix is likely vulnerable to high temperatures and is important for regular spacing of the β-strands that are connected to the β-sheet on the IBS of *Ff*IBP^15^. The formation of the disulfide bond at *Ff*IBP_CC3 is anticipated to increase the stability of the global structure as the hyperactive IBPs contain disulfide bond(s) for the intra-interaction inside of the body domain. Therefore, we generated the *Ff*IBP_CC3, which was located in the internal body domain. Finally, the most flexible region identified by MD simulation was selected as the 4th disulfide mutant (*Ff*IBP_CC4). To confirm the disulfide formation, we solved the crystal structures of the *Ff*IBP_CC1, CC2, CC3, and CC4 at 2.0, 2.1, 2.0, and 2.0 Å, respectively (Supplementary Table S1). Among these mutants, only *Ff*IBP_CC1 and *Ff*IBP_CC4 formed disulfide bonds within the intended area (Fig. 2e).

Structural superimposition indicated that wildtype and mutants are almost identical, as indicated by an RMSD value average of ~ 0.14 Å, including the orientation of the side chains throughout the structure except for the mutation regions (Supplementary Fig. S1). When the RMSD values of alpha carbon mutants were compared with the wildtype, relatively high RMSD values exceeding 0.4 Å were observed around the mutation sites of *Ff*IBP_CC2, *Ff*IBP_CC3, and *Ff*IBP_CC4. However, *Ff*IBP_CC1 did not exhibit any significant difference (Supplementary Fig. S2). Moreover, MD simulations using the *Ff*IBP_CC1 structure indicated that the disulfide bond formation between C101 and C120 stabilized the fluctuation of residues in the capping head region (Fig. 1A and Supplementary Fig. S3).

### Thermal stability of FfIBP and its derivatives

**Figure 3:**
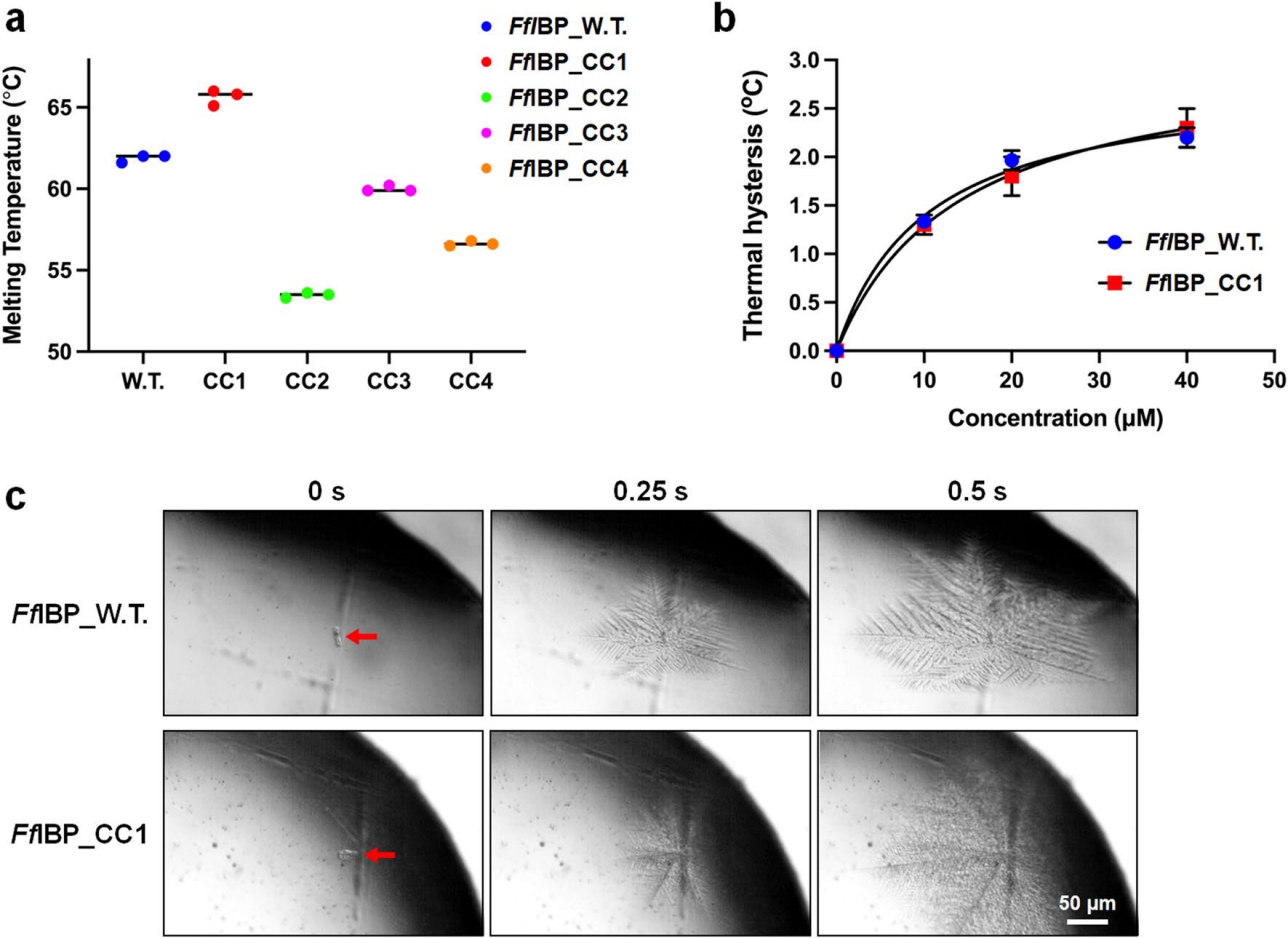
Thermal stability of mutants and thermal hysteresis (TH) activity of *Ff*IBP_WT and *Ff*IBP_CC1. (**a**) Melting temperature of *Ff*IBP, *Ff*IBP_CC1, *Ff*IBP_CC2, *Ff*IBP_CC3, and *Ff*IBP_CC4 without chemicals. (**b**) Burst ice crystal morphology in a solution of 20 µM *Ff*IBP and *Ff*IBP_CC1. Ice crystals were grown after the temperature exceeded the TH gap. (**c**) Concentration-dependent TH activity of *Ff*IBP and *Ff*IBP_CC1. All the measurements were performed in triplicate and averaged.

Given that thermostability is associated with flexible parts of the protein structure^20^ and the addition of disulfide bonds generally influences protein stability in general^21,22^, a label-free thermal stability assay on *Ff*IBP and its mutants was conducted to study protein unfolding characteristics (Supplementary Fig. S4). Consistent with our hypothesis, *Ff*IBP_CC1, which has an additional disulfide bond on the capping head region, exhibited increased thermal stability, with a melting temperature (T_m_) of 65 °C compared with wildtype *Ff*IBP (60 °C) (Fig. 3a). In contrast, *Ff*IBP_CC2 exhibited a drastic decrease in the melting temperature by approximately 8.3 °C. The mutation at the Ser96 and Lue132 to cysteines likely distorted the conformation of the residual interactions between the β helical body region and α4, which was vulnerable during thermal melting. *Ff*IBP_CC3 exhibited a ΔT_*m*_ of − 0.8 °C compared to the wildtype, which is consistent with the hypothesis that the body region is heat-insensitive (Fig. 3a). Unexpectedly, in the case of *Ff*IBP_CC4, the T_*m*_ decreased by − 5 °C, possibly due to conformational distortion caused by disulfide bond formation and a decrease in protein stability (Supplementary Fig. S1c). Thus, it is evident that introducing an additional disulfide bond in the capping read region directly or indirectly stabilizes the IBP. Therefore, only *Ff*IBP_CC1 was selected for further experiments.

### Activity of FfIBP and FfIBP_CC1

To investigate the impact of the additional disulfide bond on ice-binding properties, TH activity and ice re-recrystallization inhibition (IRI) activity assays were measured using wild-type *Ff*IBP and *Ff*IBP_CC1. TH activity was measured as described previously. Briefly, the sample was placed on a cold stage and the temperature was decreased and raised slowly to obtain a single ice crystal. The temperature gap between the melting and freezing points of the solution was considered the TH value.

**Figure 4:**
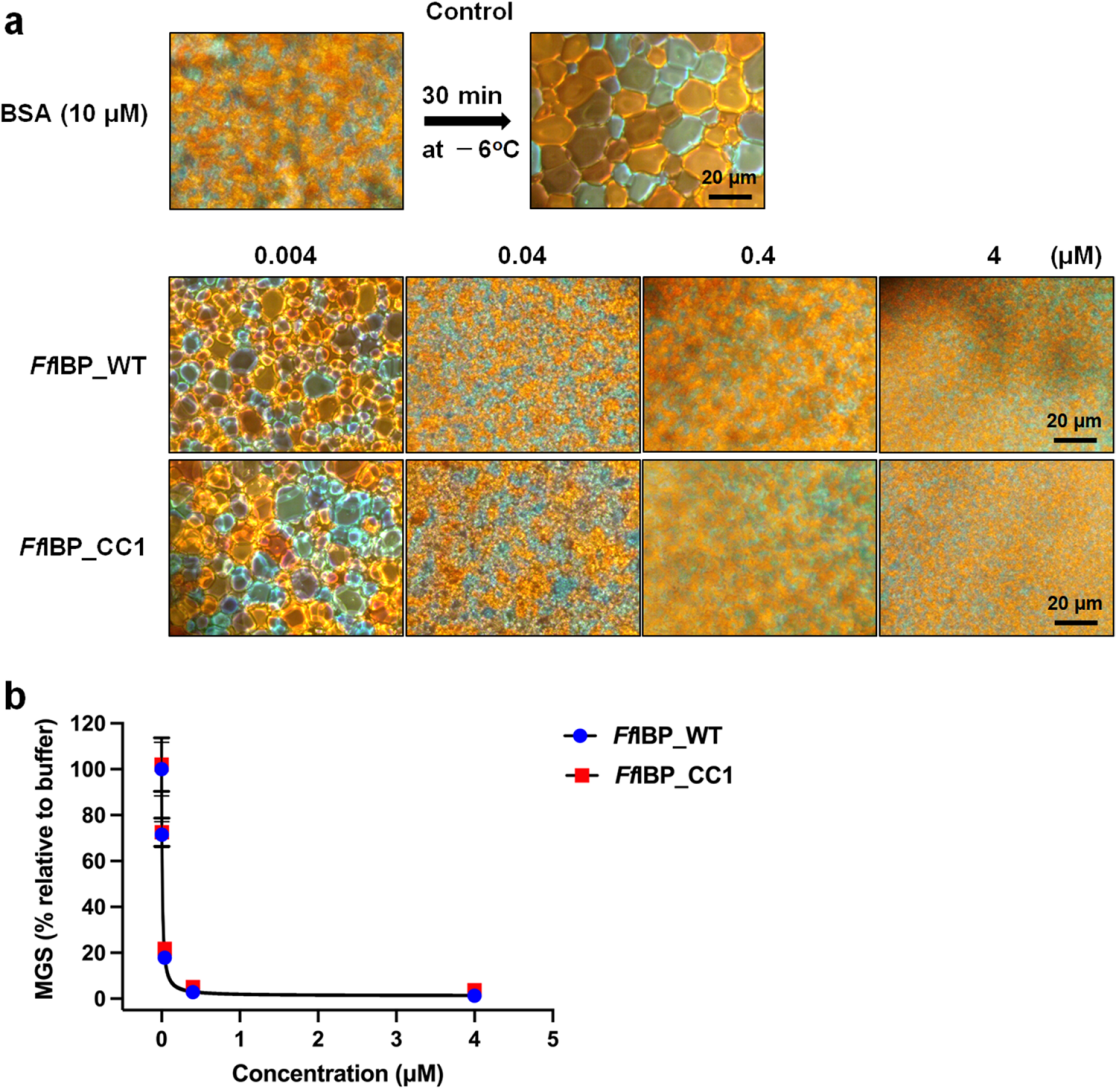
Ice recrystallization inhibition activity of *Ff*IBP and *Ff*IBP_CC1. (**a**) Morphology of growing ice crystals after incubation for 30 min. BSA protein (10 µM) was used as the negative control for the IRI assay. *Ff*IBP and *Ff*IBP_CC1 samples were measured at four different concentrations. (**b**) The graph of the concentration-dependent IRI activity of *Ff*IBP and *Ff*IBP_CC1. To determine the mean grain size (MGS) at each protein concentration, at least 30 of the largest ice grains were measured and then divided by the average size of crystals in solution without IBP.

The results indicated that *Ff*IBP_CC1 exhibited a TH value of 2.3 ± 0.2 °C at a concentration of 40 µM^15,16^. The TH activity of *Ff*IBP_CC1 was akin to that of wild-type *Ff*IBP at equivalent concentrations (Fig. 3b). Moreover, when observing the ice morphology at the freezing point, the ice busted perpendicular to the C-axis with a dendritic morphology, which was similar in the presence of both proteins in solution (Fig. 3c). Additionally, the mean grains size (MGS) values for the IRI activity of both *Ff*IBP wildtype and *Ff*IBP_CC1 were measured after a 30-min incubation at − 6 °C, called splat cooling^23^. The data indicated that *Ff*IBP_CC1 significantly inhibited the growth of ice crystals, even at a low concentration of 0.4 µM, which is comparable to that of wildtype *Ff*IBP (Fig. 4). IRI activity did not increase, even at a higher concentration of 0.4 µM IBPs. These results suggest that the additional disulfide did not interfere with the ice-binding properties of *Ff*IBP.

### Stability of IBPs

**Figure 5:**
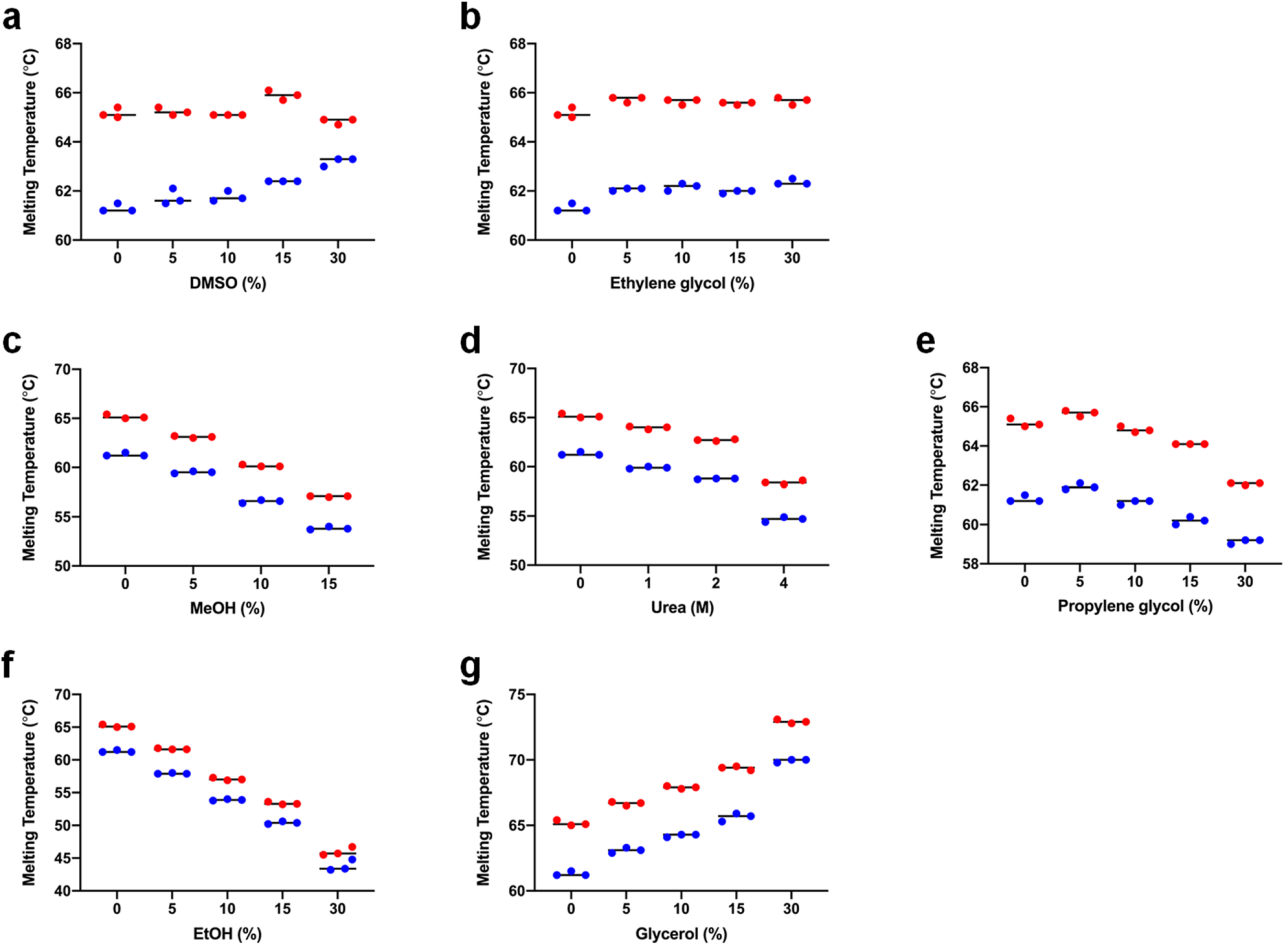
Thermal shift assay of *Ff*IBP and *Ff*IBP_CC1 in various chemicals. Melting temperature (Tm) of *Ff*IBP and *Ff*IBP_CC1 as a function of the concentration of DMSO (**a**), ethylene glycol (**b**), methanol (**c**), Urea (**d**), propylene glycol (**e**), ethanol (**f**), and glycerol (**g**) was determined at emission wavelengths of 330 and 350 nm using the NanoTemper Tycho NT.6. Triplicate measurements (red or blue dots) were averaged and marked with a black line. DMSO, dimethyl sulfoxide.

DMSO, glycerol, ethylene glycol, methanol, and 1,2-propanediol are well-recognized CPAs^13,24^. However, these compounds also negatively affect proteins, potentially causing denaturation, which can result in the loss of protein activity and function. Chemical stability analysis of wild-type *Ff*IBP and *Ff*IBP_CC1 with the mentioned chemicals demonstrated that *Ff*IBP_CC1 still maintained a high melting temperature (T_*m*_), indicating that disulfide bond formation remained stable and was effective in preserving protein stability in the presence of these chemicals (Fig. 5).

These chemicals can be categorized into three groups based on their melting temperatures. In group A, which included DMSO and ethylene glycol, the T_*m*_ of *Ff*IBP_CC1 remained relatively unchanged, even with an increase in the concentration of the chemicals, implying that the IBPs remained stable even with 30% of the chemicals in group A. In group B, which contained methanol, urea, and propylene glycol, the melting temperature patterns with increased chemical concentrations were similar, and the gaps in the melting temperatures of the two proteins were maintained. The last group, consisting of ethanol and glycerol, exhibited drastic changes in the melting temperatures in concert with an increased chemical concentration. Notably, glycerol was the only chemical that increased the melting temperature of both *Ff*IBP and *Ff*IBP_CC1. These findings indicate that the disulfide bond formation remains intact in IBPs in various protein denaturants and enhances the chemical stability of *Ff*IBP and *Ff*IBP_CC1. Furthermore, to assess the stability of IBPs over a broad pH range, the CD was used to monitor changes in the secondary structure under different pH conditions and incubation was extended at low temperatures. The results revealed that the secondary structures of both IBPs remained unchanged from pH 4.0 to pH 9.0, and 7 days of incubation at 0 °C, indicating that the *Ff*IBP and *Ff*IBP_CC1 are resistant to a wide pH range and low temperatures (Supplementary Fig. S5).

### IRI activity in the presence of CPAs

**Figure 6:**
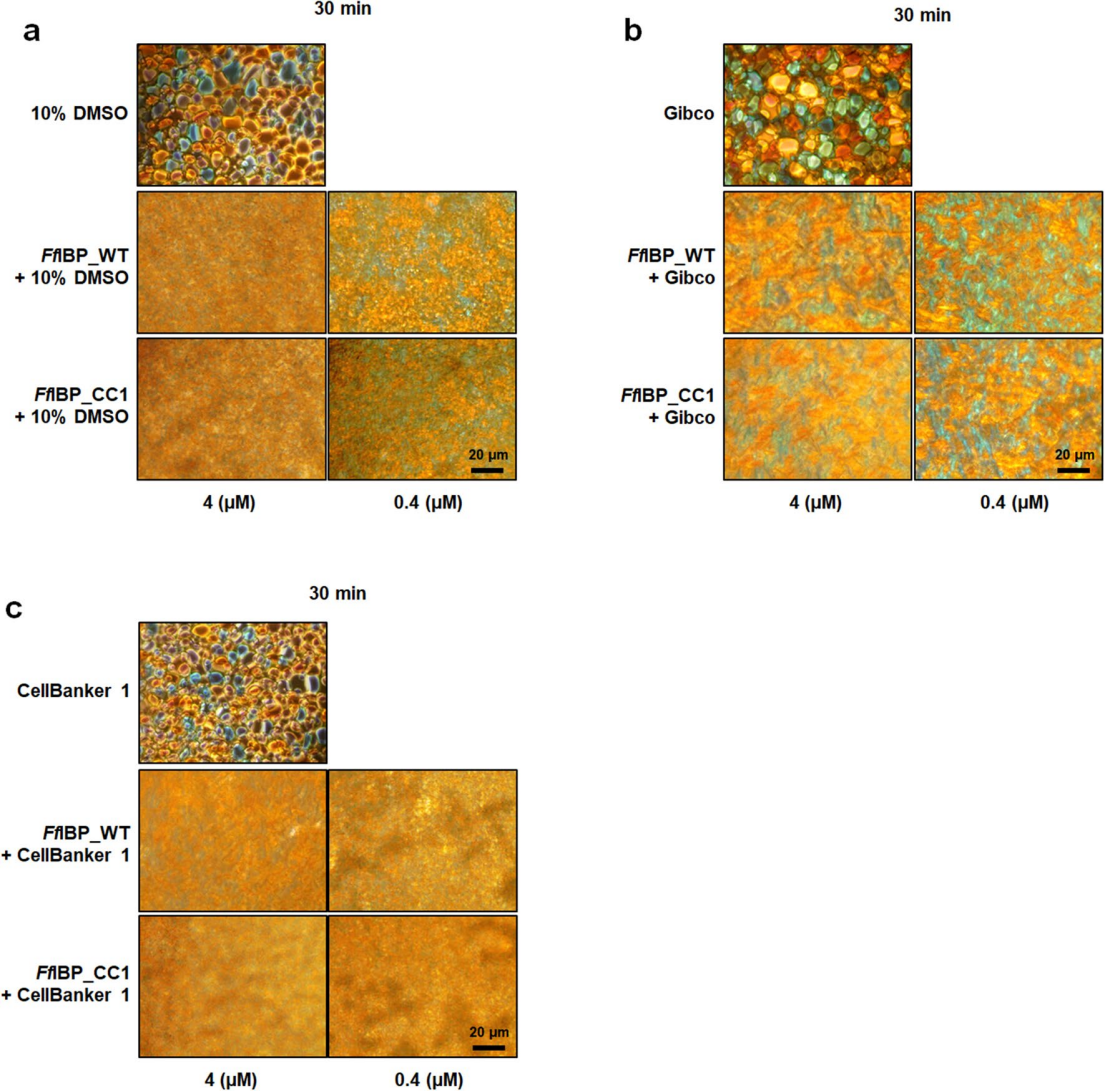
Assessment of ice recrystallization inhibition (IRI) activity in the presence of CPAs. (**a**) *Ff*IBP and *Ff*IBP_CC1 samples were measured at concentrations of 0.4 µM or 4 µM in 10% DMSO after 30 min of incubation at − 8 °C, with 10% DMSO used as the negative control. Commercial cryoprotectants Gibco™ Synth-a-Freeze™ Cryopreservation Medium (**b**) and CellBanker 1(**c**) were also evaluated for measuring IRI activity. Scale bars, 20 μm.

To assess whether the ice-binding properties of *Ff*IBP and *Ff*IBP_CC1 were retained in the presence of CPAs, the IRI activity was measured in the presence of DMSO 10%, Gibco, CellBanker, and commercially available CPAs (Fig. 6). The minimum protein concentration showing IRI activity (0.4 µM) and a 4 µM concentration was mixed with the chemicals and incubated for 30 min at − 8 °C. The results demonstrated that the IRI activity of *Ff*IBP and *Ff*IBP_CC1 remained effective at a concentration of 0.4 µM and 4 µM in 10% DMSO. Moreover, in the presence of the Gibco and CellBanker, *Ff*IBP and *Ff*IBP_CC1 showed similar IRI activity at a concentration of 0.4 µM, indicating that the ice-binding property of *Ff*IBP, as well as that of *Ff*IBP_CC1, is functional in the CPAs.

## Discussion

The introduction of a new disulfide bond could lead to a reduction or loss of enzymatic activity because of interference with the precise stereochemistry or dynamic movement of residues in the active site^25^. However, this approach has been used in protein engineering to enhance protein stability and prevent thermal damage^21,22,26^. AFPs and IBPs interact with solid ice, and the rigidity or stability of IBPs has been suggested as a critical factor in extending the binding time and increasing the strength of the bond between the protein and ice^16^. In this context, engineering disulfide bonds in IBPs can be a useful strategy to generate stable and functional analogs and understand the structure–activity relationships of IBPs.

To develop a mutant IBP with improved TH activity and thermal stability, we first identified the structurally unstable regions of *Ff*IBP by denaturation of the secondary structure and MD simulations. We designed a mutant IBP by introducing additional disulfide bonds to stabilize these regions. Four sets (*Ff*IBP_CC1, *Ff*IBP_CC2, *Ff*IBP_CC3, and *Ff*IBP_CC4) of mutation sites for disulfide bond formation in the flexible region of the *Ff*IBP were introduced. Successful formation of the disulfide bond at the intended location in *Ff*IBP_CC1 and *Ff*IBP_CC4 was confirmed by X-ray crystallography, whereas the rest did not form a disulfide bond. The failure to form a disulfide bond may be due to dihedral angle constraints and the inappropriate distance between the two cysteines. Contrary to our expectations, *Ff*IBP_CC4 experienced a decrease of 5 °C compared to the WT form. The slight side chain rotation of the C176, observed in the structural comparison, does not explain this destabilization. However, we postulate that the forced angle change due to the disulfide bond negatively affects neighboring residues involved in the hydrophobic core, such as Trp174, Leu178, and Val198 (Supplementary Figure S1). It is worth noting that the initial 350 nm/330 nm ratio for *Ff*IBP_CC4 is lower than that of other mutants, including the wild-type (Supplementary Figure S4). This difference may be attributed to the effect of disulfide formation on Trp174 in *Ff*IBP_CC4. A similar destabilization upon disulfide bond formation was observed in the titin polyprotein^27^. These findings highlight the importance of selecting the appropriate cysteine mutant residues to facilitate disulfide bond formation and enhance thermal stability in IBPs. In addition, compared to the wildtype *Ff*IBP, only mutant *Ff*IBP_CC1 exhibited an improvement in thermal stability by 5 °C.

The stability increase was also observed in the thermal stability measured by CD. *Ff*IBP_CC1 exhibited greater thermal stability by about 3 °C in comparison to wildtype CD spectra (Supplementary Figure S6). However, the melting temperature of *Ff*IBP measured by CD spectra was only 47.8 °C, whereas Tycho showed it to be 60 °C. Initially, we speculated that the salt increases the protein stability because we removed only NaCl in the optimized buffer condition for CD, and many proteins showed salt concentration-dependent stability. However, Tm data at different salt concentrations showed no significant difference (Supplementary Figure S7). Next, we hypothesized that this discrepancy could be due to the difference in measurement methods. CD spectra used 210 nm wavelength to calculate the Tm value, which reflects the helix structure of the protein^28^, whereas Tycho measured the ratio of tryptophan fluorescence at 350 and 330 nm. The three tryptophans are located inside the β-helical body region of *Ff*IBP. Moreover, this indicates that the conformational/unfolding transition of *Ff*IBP is initiated in the helix region, and the β-helical body region is the last step in the unfolding process at higher temperature.

Interestingly, unlike wildtype, *Ff*IBP_CC1 underwent a two-step denaturation. The initial denaturation was similar to that of *Ff*IBP until 66 °C. When the temperature exceeded 68 °C, the spectra of delta epsilon tended to be flattened. The secondary structure prediction indicated that the α-helices (Helix1) were completely denatured, and the β-sheets were reduced beyond this temperature (Supplementary Figure S6b). Although further research is required to determine the detailed denaturation process, it is believed that the disulfide bond could affect the protein’s conformational changes during temperature increases.

In addition, *Ff*IBP_CC1 exhibited increased thermal stability, even when exposed to high concentrations of chemical denaturants such as DMSO, MeOH, Urea, propylene glycol, EtOH, and glycerol. Usually, chemical denaturants exhibit a high affinity for water and disrupt the hydrogen bonds or hydrophobic interactions that stabilize the folded conformation of the protein by attacking the flexible local areas of the proteins^29^. Thus, disulfide bond formation in *Ff*IBP likely contributes to minimizing the structural perturbations induced by these chemicals and heat inputs. Moreover, *Ff*IBP and *Ff*IBP_CC1 exhibited effective IRI activity in the presence of DMSO 10%, Gibco, and CellBanker, which are commercial cryoprotectants, indicating that the proteins were intact and functional in these chemicals.

The structural stability of proteins is greatly influenced by non-covalent bonding, including hydrogen bonds, electrostatic interactions, and hydrophobicity. Among these, the hydrophobic effect within the protein core appears to be a crucial factor for protein stability^30^, as evidenced by studies such as the methyl introduction study^31^ that aimed to increase protein thermal stability and the comparison between mesophilic and thermophilic proteins^32^. The addition of disulfide bonds to proteins has been observed to decrease thermal unfolding and denaturation by increasing the activation energy barrier to the unfolded states, thus protecting the hydrophobic core^30^. Moreover, the hydrophobic effect is suggested to be also mainly associated with the cold denaturation of proteins^33^. Therefore, disulfide bond formation in *Ff*IBP (*Ff*IBP_CC1) may also contribute to resistance to cold denaturation, although we did not observe the loss of both *Ff*IBP and *Ff*IBP_CC1 during freezing–thawing repetition (Supplementary Figure S4). Nevertheless, we anticipate that *Ff*IBP_CC1 would exhibit beneficial effects on cell protection as a cryoprotective agent during the cryopreservation process because of the improved chemical stability^34^.

Taken together, our findings suggest that *Ff*IBP is a resilient protein that maintains its ice-binding properties, even in the presence of denaturants. Furthermore, the incorporation of disulfide bonds into the capping head region of *Ff*IBP enhances its stability and resistance to thermal and chemical denaturation. Given that *Ff*IBP shares a similar structure with DUF3494 IBPs, the most widespread IBP family, our strategy can be applied to engineer other IBPs for use as natural cryopreservation agents^35^. Overall, this study provides new insights into the physicochemical stability of IBPs and suggests novel approaches for enhancing their stability.

## Methods

### Site-directed mutagenesis

Desired mutants were generated using site-directed mutagenesis. The primers used for mutagenesis are listed in Supplementary Table S2. The mutated sequences were confirmed via DNA sequencing.

### Overexpression and purification of FfIBP and mutants

The constructs of pColdI-FfIBP mutants were transformed into *Escherichia coli* Rosetta-gamiTM 2 (DE3) (Novagen, Darmstadt, Germany) and grown in Luria–Bertani (LB) medium containing ampicillin 100 µg/mL. The culture was initially grown at 37 °C until the optical density at 600 nm reached 0.6. Subsequently, the temperature was lowered to 15 °C, and the culture was induced with 0.5 mM IPTG for protein expression. After a 72-h incubation, the cells were harvested, resuspended, and lysed using a sonication buffer (30 mM Tris–HCl pH 8.5, 150 mM NaCl, and 5 mM imidazole). The supernatant was separated via centrifugation and loaded onto a pre-equilibrated Ni–NTA resin agarose column (Qiagen, Hilden, Germany). The bound protein was eluted and treated with factor Xa protease (New England Biolabs, Ipswich, MA, USA) to remove the 6×His tag. Subsequently, proteins were purified using a Superdex 200 prep-grade column (GE Healthcare, Chicago, IL, USA). The purified protein was collected and concentrated in a buffer consisting of 30 mM Tris–HCl (pH 8.5 and 300 mM NaCl using an Amicon Ultra Centrifugal Filter (Merck, Darmstadt, Germany).

### Crystallization and data collection

The crystallization conditions for *Ff*IBP mutants were obtained from a previous report^36^, which was originally used for wild-type *Ff*IBP. The hanging-drop vapor diffusion method was employed by mixing 1.5 μL of the protein solution (approximately 20 mg/mL for each protein) with 1.5 μL of the crystallization solution (0.1 M sodium citrate pH 4.4, 3 M sodium chloride). X-ray diffraction datasets were collected at the BL-5C beamline of the Pohang Light Source operated by the Pohang Accelerator Laboratory in Pohang, Republic of Korea. XDS software was used for indexing, integrating, and scaling the dataset^37^.

### Structure determination and refinement

The MOLREP program from the CCP4i suite was used to determine the crystal structure of the mutants through a molecular replacement method using the wildtype *Ff*IBP structure as a search model. The structure was refined using a combination of the REFMAC5 and Phenix. Refine software. MolProbity was used to check model quality^38^, and all structural representations were generated using PyMOL^39^. The mutant coordinates and structural factors were deposited in the Protein Data Bank RCSB under the accession codes 8X0Z, 8X1L, 8X1O, and 8X1P. Detailed data collection and refinement statistics are provided in Supplementary Table S1.

### MD simulation

Protein structures were subjected to MD simulations using GROMACS 2022 with the CHARMM Force Field^40,41^. The process and syntax are referred to in the GROMACS documentation. After the removal of water molecules from the crystallography, the *Ff*IBP structure was used as the initial coordinate. The structure was located in the center of the cubic system, solvated with a TIP4P water model, and neutralized with 0.15 M sodium chloride. The sides of the cubic box had dimensions of 15 nm (3,375,000 Å^3^). One thousand steps of steepest descent (SD) followed by 50,000 steps of the Adopted Basis Newton–Raphson (ABNR) algorithms were applied for minimization. After 100 ps of isochoric (NVT) and isobaric isothermal equilibration (NPT), the MD simulation was performed at 300 K with a 2 fs time step for 100 ns in an NPT ensemble under periodic boundary conditions (PBC) in all three dimensions. The long-range electrostatic interactions were calculated using the particle-mesh Ewald (PME) method^42^. The RMSD, RMSF, and distances between residues from the trajectory files were analyzed using GROMACS. Pymol^39^ and VMD^43^ programs were used for analysis and figure generation.

### TH activity

The TH activity of the IBPs was measured as previously described^15,36^. The sample was placed in the holes of an aluminum disk on the stage and quickly frozen at approximately − 40 °C using a nanoliter osmometer (Otago Osmometers, Dunedin, New Zealand). The temperature was slowly increased until only one ice crystal remained (melting temperature). Subsequently, the temperature was slowly lowered again (at a rate of ~ 0.5 °C per min) until the ice crystal started to grow (freezing temperature). The shape of the ice crystals at the freezing point was observed under a microscope, and photographs were taken using a Canon Power Shot 620. The measurements were performed in a buffer containing 30 mM Tris–HCl (pH 8.5) and 150 mM NaCl at various sample concentrations.

### IRI assay

IRI was measured using a modified “splat cooling” method^23^. Protein samples were diluted to the desired concentration in a buffer containing 30 mM Tris–HCl (pH 8.5) and 150 mM NaCl. A 10 µL of the droplet was released from a height of 1.20 m onto a 16 mm glass coverslip resting on a polished aluminum surface that was situated on a pool of liquid nitrogen. Upon impact with the cold coverslip, the droplet instantaneously froze into a wafer-like structure with a diameter of approximately 12 mm and a thickness of 10 µm. The coverslip with the frozen sample was then placed on a Linkam TMHS6000 cold stage (Linkam Scientific Instruments, Surrey, UK) and held at − 6 °C or − 8 °C for 30 min. The IRI assay was performed using an Olympus BX51 microscope equipped with crossed polarizers, and images were captured using an Olympus DP71 CCD camera. The IRI activity was evaluated by measuring the largest dimension of individual ice grains in various fields of view. The MGS was calculated by averaging the values of the 30 largest ice grains. A smaller MGS indicates a higher level of IRI activity, as it indicates the effective prevention of ice crystals from growing during the constant continuous process at − 6 °C or − 8 °C.

### Thermal shift assay

Label-free thermal shift assays were performed using a Tycho NT.6 instrument (NanoTemper Technologies GmbH, Munich, Germany). Protein samples were diluted to a concentration of 1 mg/mL in a buffer containing 20 mM Tris–HCl at pH 8.0. Following the incubation, the samples were heated in a glass capillary from 35 to 95 °C at a rate of 30 °C per min. During this process, the intrinsic fluorescence emanating from tryptophan and tyrosine residues was recorded at wavelengths of 330 and 350 nm, respectively. Subsequently, the ratio of fluorescence at 350/330 nm and the melting temperature were calculated using the internal evaluation capabilities of a Tycho instrument. The data presented in this study represent the results of three independent experiments.

### Circular dichroism spectroscopy to analyze the unfolding of IBP

CD spectroscopy was performed using a Chirascan CD spectrometer (Applied Photophysics Co., Leatherhead, UK) and a Peltier temperature controller. Protein samples (0.1 mg/mL) in 10 mM Tris–HCl (pH 8.5) were loaded into quartz cuvettes with a path length of 1 mm (Hellma, Plainview, NY, USA), and the absorbance was measured from 190 to 250 nm with 0.5 nm steps and 0.5 s/step at a 1 mm path length. The temperature was set from 20 to 90 °C with a 2 °C increment per step. The measurements were repeated three times, and the buffer spectra were subtracted from the average spectra to obtain the final trace. The analyzed data were obtained using the interactive circular dichroism analysis software CDNN^28,44^ and expressed as the Delta epsilon, given in M^−1^ cm^−1^. The proportion of α-helix, β-strand, turn, and other (mostly coil) structures at different temperatures was calculated using BeStSel software^45^.

### Stability of IBPs at different pHs and low temperature

To evaluate the impact of various pH levels on the stability of *F*fIBP, its structural denaturation profile was determined using CD within a pH range of 4–9. At pH 4 and 5, *F*fIBP and *F*fIBP_CC1 were diluted in sodium acetate buffer (10 mM sodium acetate buffer); for pH 6 and 7, sodium phosphate buffer (10 mM sodium phosphate buffer) was used; for pH 8 and 9, Tris–HCl buffer (10 mM Tris–HCl) was used.

To determine the reversibility of cold denaturation, *F*fIBP and *F*fIBP_CC1 were incubated at 0 °C for 1, 2, 3, and 7 days. Following each incubation period, the proteins were centrifuged at 16,000×*g* for 10 min, and the supernatant was analyzed using CD to evaluate their stability^46^. In both experiments conducted under various pH conditions and at low temperatures, proteins were used at a final concentration of 0.1 mg/mL and assessed by scanning at 190–250 nm as described above.

### CPA information

Two commercial CPAs were examined to determine their impact on protein stability and functionality: Gibco™ Synth-a-Freeze™ Cryopreservation Medium (Gibco, Thermo Fisher Scientific, Waltham, MA, USA) and CELLBANKER 1 (Amsbio Inc., Cambridge, M). Gibco™ Synth-a-Freeze™ Cryopreservation Medium is employed for the cryopreservation of a wide variety of mammalian cells, including keratinocyte cell, embryonic stem cell, neural stem cell, and mesenchymal stem cell. CELLBANKER 1 is designed for a broad spectrum of mammalian cells, such as CHO, Jurkat, CEM, and K562. Both media contain 10% DMSO as cryoprotectant.

### Supplementary Information

Supplementary Information.

## Data Availability

The structure datasets generated during the current study are available in the RCSB PDB Protein Data Bank repository. The PDB accession numbers are as follows: 8X0Z for *Ff*IBP_CC1, 8X1L for *Ff*IBP_CC2, 8X1O for *Ff*IBP_CC3, and 8X1P for *Ff*IBP_CC4.
