# Supplementary material for: Engineered ice-binding protein (FfIBP) shows increased stability and resistance to thermal and chemical denaturation compared to the wildtype
Source: Sci Rep. 2024 Feb 8;14:3234. doi: 10.1038/s41598-024-53864-w (PMC10853241; doi:10.1038/s41598-024-53864-w)
Supplement: Supplementary file 1 — Supplementary Information. [file 41598_2024_53864_MOESM1_ESM.docx]

**Supplementary Material**

**Engineered ice-binding protein (*Ff*IBP) shows increased stability and resistance to thermal and chemical denaturation compared to the wildtype**

**Authors:** Yewon Nam, Dieu Linh Nguyen, Trang Hoang, Bogeun Kim, Jun Hyuck Lee, Hackwon Do

**Table of Contents**

**Supplementary Table S1.** X-ray diffraction and refinement statistics.

**Supplementary Table S2.** Primers used in this study.

**Supplementary Figure S1.** Superimposed crystal structures of wild-type and *Ff*IBP_CC1 mutant proteins.

**Supplementary Figure S2.** Diagram of RMSD per residue for mutant structures compared with wild-type *Ff*IBP.

**Supplementary Figure S3.** Distance between residues 101 and 120 during the MD simulation.

**Supplementary Figure S4.** Denaturation profiles of *Ff*IBP and mutants in 20 mM Tris-HCl and 150 mM NaCl during temperature increase.

**Supplementary Figure S5.** Circular dichroism (CD) spectra of *Ff*IBP and *Ff*IBP_CC1 were recorded at different pH levels and at various time points during incubation at 0°C.

**Supplementary Figure S6.** Circular dichroism (CD) spectroscopy of *Ff*IBP_CC1 and comparison with *Ff*IBP during the unfolding process.

**Supplementary Figure S7.** Thermal stability assay at different salt (NaCl) concentrations using Tycho NT.6.

**Supplementary Table S1.** X-ray diffraction data collection and refinement statistics.

| Data set | *Ff*IBP_CC1 | *Ff*IBP_CC2 | *Ff*IBP_CC3 | *Ff*IBP_CC4 |
| --- | --- | --- | --- | --- |
| X-ray source | BL-5C beamline | BL-5C beamline | BL-5C beamline | BL-5C beamline |
| Space group | *P*4_1_22 | *P*4_1_22 | *P*4_1_22 | *P*4_1_22 |
| Unit-cell parameters (Å, °) | a=b=69.23, c=178.42, α=β=γ= 90 | a=b=69.06, c=178.35, α=β=γ= 90 | a=b=69.14, c=178.21, α=β=γ= 90 | a=b=69.04, c=178.57, α=β=γ= 90 |
| Wavelength (Å) | 1.0 | 1.0 | 1.0 | 1.0 |
| Resolution (Å) | 29.25–2.10 (2.16–2.10) | 29.86–2.00 (2.05–2.00) | 29.21–2.10 (2.16–2.10) | 28.82–2.00 (2.05–2.00) |
| Total reflections | 669,488 (57,473) | 776,169 (59,545) | 664,463 (57,738) | 775,282 (59,176) |
| Unique reflections | 26,223 (2,099) | 30,097 (2,183) | 26,115 (2,102) | 30,121 (2,185) |
| Average I/σ (I) | 39.2 (8.8) | 31.9 (5.0) | 35.7 (8.9) | 35.7 (8.9) |
| *R*_merge_^a^ | 0.08 (0.64) | 0.06 (0.70) | 0.06 (0.40) | 0.04 (0.16) |
| Redundancy | 25.5 (27.4) | 25.8 (27.3) | 25.4 (27.5) | 25.7 (27.1) |
| Completeness (%) | 100.0 (100.0) | 100.0 (100.0) | 100.0 (100.0) | 100.0 (100.0) |
| Refinement |  |  |  |  |
| Resolution range (Å) | 29.25–2.10 (2.16–2.10) | 29.88–2.00 (2.05–2.00) | 29.23–2.10 (2.16–2.10) | 28.84–2.00 (2.05–2.10) |
| No. of working set reflections | 26,216 (2,684) | 28,613 (2,064) | 24,790 (1,765) | 28,638 (2,067) |
| No. of test set reflections | 1,328 (141) | 1,484 (119) | 1,324 (100) | 1,482 (117) |
| No. of atoms | 1,554 | 1,552 | 1,550 | 1,761 |
| No. of water molecules | 159 | 103 | 142 | 213 |
| *R*_cryst_^b^ | 0.25 (0.28) | 0.22 (0.32) | 0.24 (0.27) | 0.24 (0.27) |
| *R*_free_^c^ | 0.26 (0.33) | 0.25 (0.33) | 0.28 (0.35) | 0.26 (0.27) |
| r.m.s. bond length (Å) | 0.007 | 0.012 | 0.013 | 0.013 |
| r.m.s. bond angle (°) | 0.931 | 1.632 | 1.631 | 1.631 |
| Average B value (Å^2^) (protein) | 37.36 | 43.43 | 39.17 | 28.25 |
| Average B value (Å^2^) (solvent) | 43.51 | 46.29 | 44.22 | 38.49 |
| Ramachandran plot |  |  |  |  |
| Favored (%) | 97.2 | 94.4 | 95.8 | 94.8 |
| Allowed (%) | 2.8 | 4.6 | 2.8 | 3.8 |
| Outliers (%) | 0.0 | 1.4 | 1.4 | 1.4 |

^a^ *R*_merge_ = ∑｜<I> - I｜/∑<I>.

^b^ *R*_cryst_ = ∑｜|Fo| - |Fc|｜/∑|Fo|.

^c^ *R*_free_ calculated with 5% of all reflections excluded from refinement stages using high-resolution data.

The values in parentheses refer to the highest-resolution shells.

**Supplementary Table S2.** Primers used in this study.

| Site of mutation | | Sequences | |
| --- | --- | --- | --- |
| *Ff*IBP_CC1 | A101C | Forward | GCAAGTCCAATTACAGGATGCGCTATTCTTTTA |
|  |  | Reverse | TAAAAGAATAGCGCATCCTGTAATTGGACTTGC |
|  | A120C | Forward | CATATTTTCAGTTGATGCTTGCGGACCTGCTTGC |
|  |  | Reverse | GTTATTTTGCAAGCAGGTCCGCAAGCATCAACTG |
| *Ff*IBP_CC2 | S96C | Forward | GGTGATGTTGGTGCATGCCCAATTACAGGAGCC |
|  |  | Reverse | GGCTCCTGTAATTGGGCATGCACCAACATCACC |
|  | L132C | Forward | CTGATGCTTCACGTTGCACTACAGCTGTAGGTG |
|  |  | Reverse | CATGTCACCTACAGCTGTAGTGCAACGTGAAGC |
| *Ff*IBP_CC3 | M249C | Forward | GTCAAACTGGTATAAATTGCAAAACAGCCGC |
|  |  | Reverse | GCGGCTGTTTTGCAATTTATACCAGTTTGAC |
|  | N270C | Forward | GCAGTTACACTACAAATGTGCACCGTTACCATACC |
|  |  | Reverse | GGTATGGTAACGGTGCACATTTGTAGTGTAACTGC |
| *Ff*IBP_CC4 | G160C | Forward | TAAATTTAGGGGCTTGCACTATCGGTGGA |
|  |  | Reverse | TTTCCACCGATAGTGCAAGCCCCTAAATT |
|  | S176C | Forward | CCAGGTTTATATAAATGGACATGCACATTAAACATCCC |
|  |  | Reverse | AGGGATGTTTAATGTGCATGTCCATTTATATAAACCTGG |

**
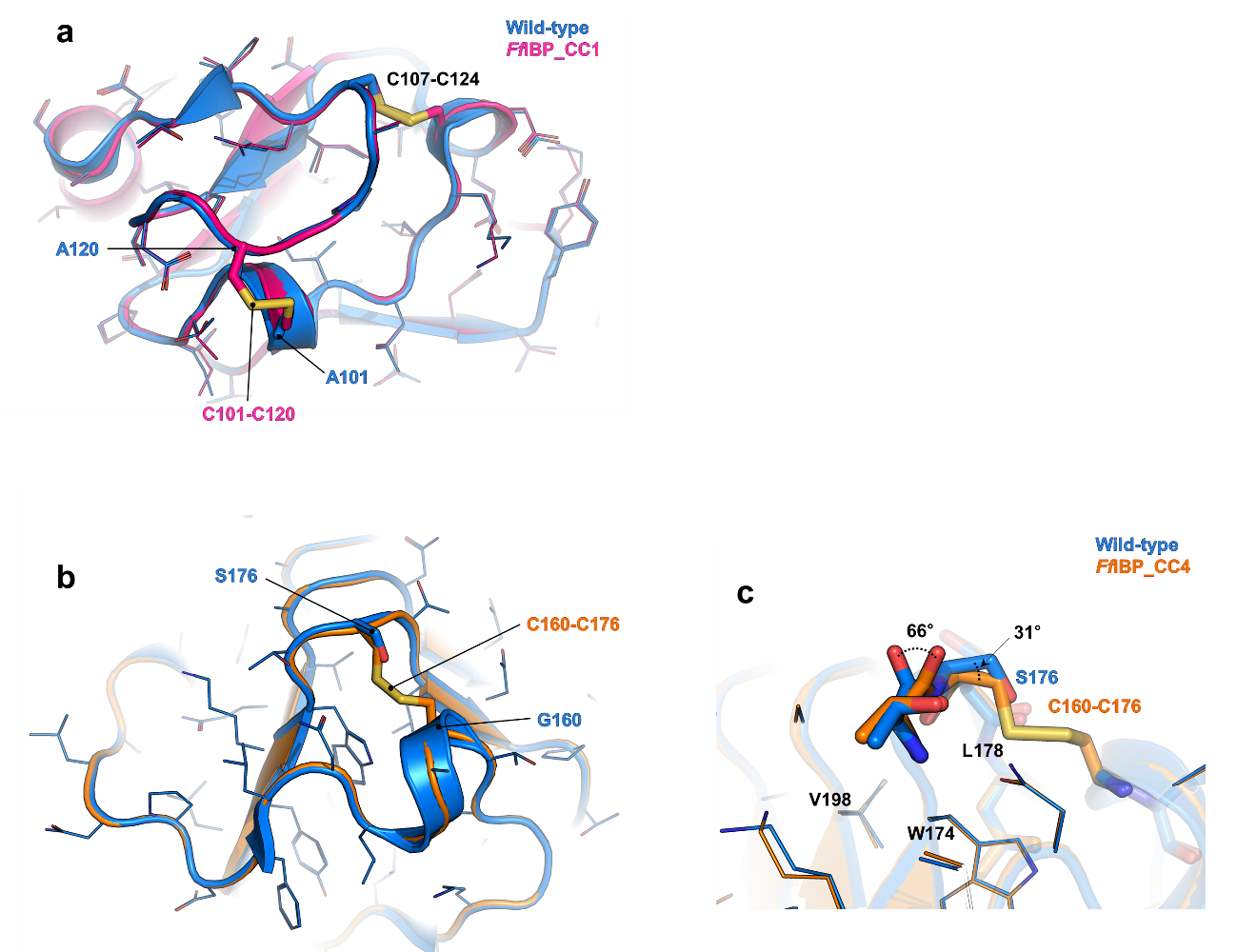
Supplementary Figure S1.** Superimposed crystal structures of wild-type, *Ff*IBP_CC1 (**a**, cyan), and *Ff*IBP_CC4 mutants (**b** and **c**, magenta). (**a**) Ala101 and Ala120 in the wild type and the disulfide bond of *Ff*IBP_CC1 are indicated by sticks. (**b** and **c**) Mutation site of *Ff*IBP_CC4 and superimposition with the wild-type. The formation of disulfide bonds in *Ff*IBP_CC4 caused torsion of the peptide backbone. Both mutants were almost identical to the WT, with RMSD values of 0.135 and 0.143, respectively.

**
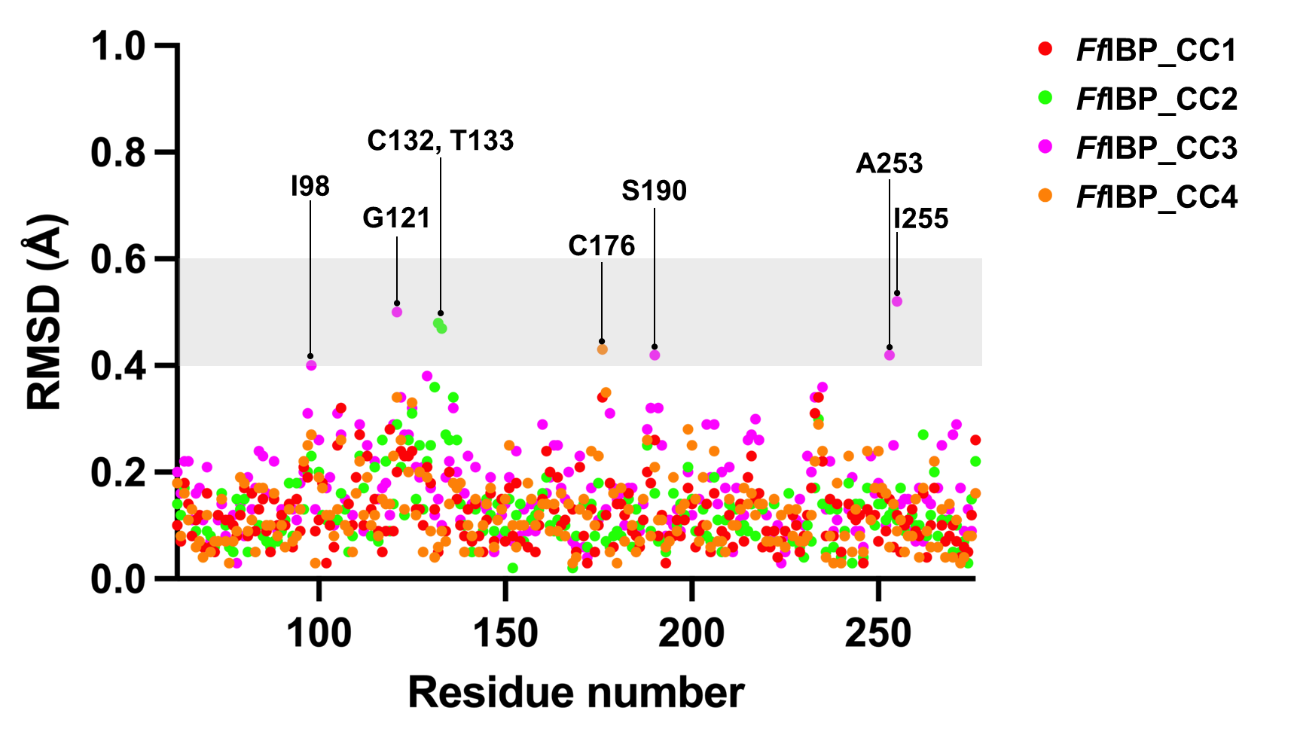
**

**Supplementary Figure S2.** Diagram of RMSD per residue for mutant structures compared with wild-type *Ff*IBP. The RMSD value per residue for each mutant is indicated by a different color. The RMSD values of between 0.4 and 0.6 Å are shaded with grey. *Ff*IBP, engineered ice-binding protein; RMSD, root mean square deviation.

**
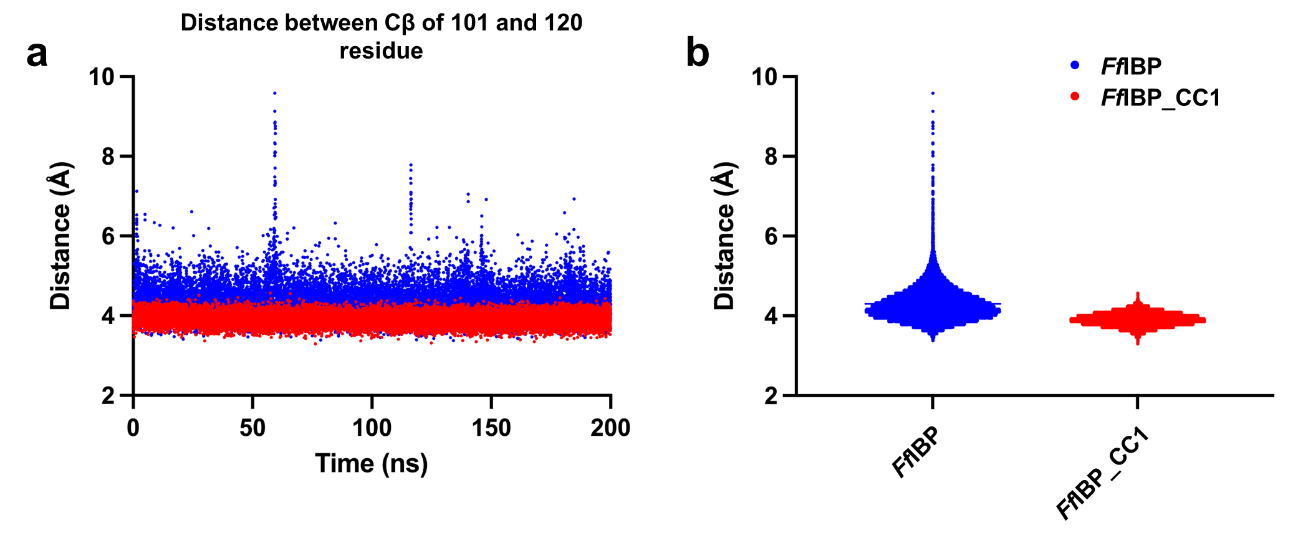
**

**Supplementary Figure S3.** Distance between residues 101 and 120 during the MD simulation. (**a**) Distance plots between residues in *Ff*IBP (Ala101, atom type Cβ; Ala120, atom type Cβ) and *Ff*IBP_CC1 (Cys101, atom type Cβ; Cys120, atom type Cβ) from independent simulation runs. (**b**) Histograms of the distance between the two residues shown in **a**. *Ff*IBP, engineered ice-binding protein; MD, molecular dynamics.

**
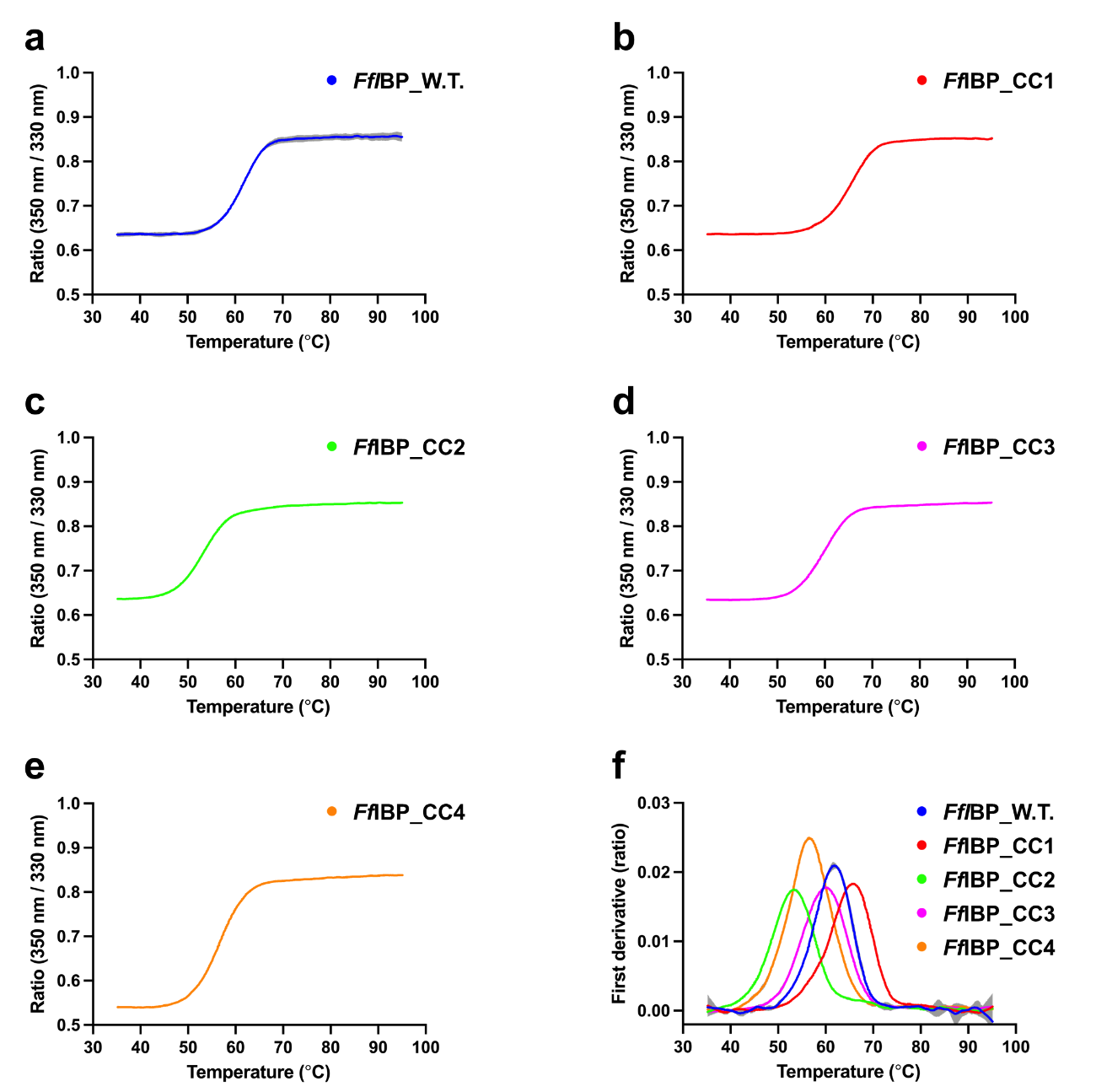
Supplementary Figure S4.** Denaturation profiles of *Ff*IBP and mutants in 20 mM Tris-HCl and 150 mM NaCl during temperature increase. Fluorescence intensities for *Ff*IBP (**a**), *Ff*IBP_CC1 (**b**), *Ff*IBP_CC2 (**c**), *Ff*IBP_CC3 (**d**), and *Ff*IBP_CC4 (**e**) were measured at 350/330 nm ratio using Tycho NT.6 instrument and represented in different color codes. Tm values for each proteins were calculated as the inflection point of each curve. (**f**) The corresponding first derivatives from Figure S4a to Figure S4e are displayed using the same color code. The shaded area represents upper and lower error limits.

**
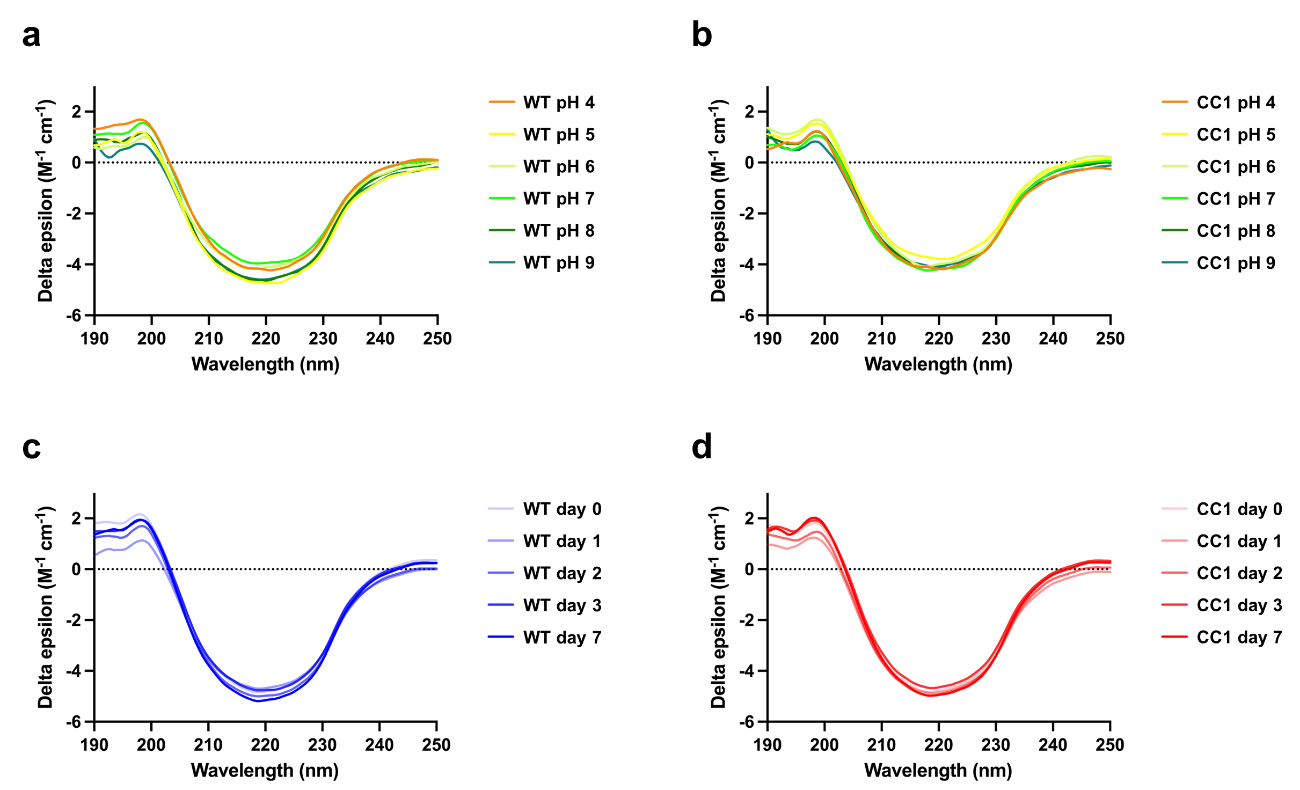
Supplementary Figure S5.** The circular dichroism (CD) spectra of *Ff*IBP and *Ff*IBP_CC1 were recorded at different pH levels and at various time points during incubation at 0°C. (**a**, **b**) Far-UV CD spectroscopic analysis of *Ff*IBP and *Ff*IBP_CC1 was conducted at pH values ranging from 4 to 9. (**c**, **d**) Protein solution samples were incubated at 0°C, centrifuged at 16,000 x g for 10 minutes, and the supernatant was analyzed using CD to assess their stability.


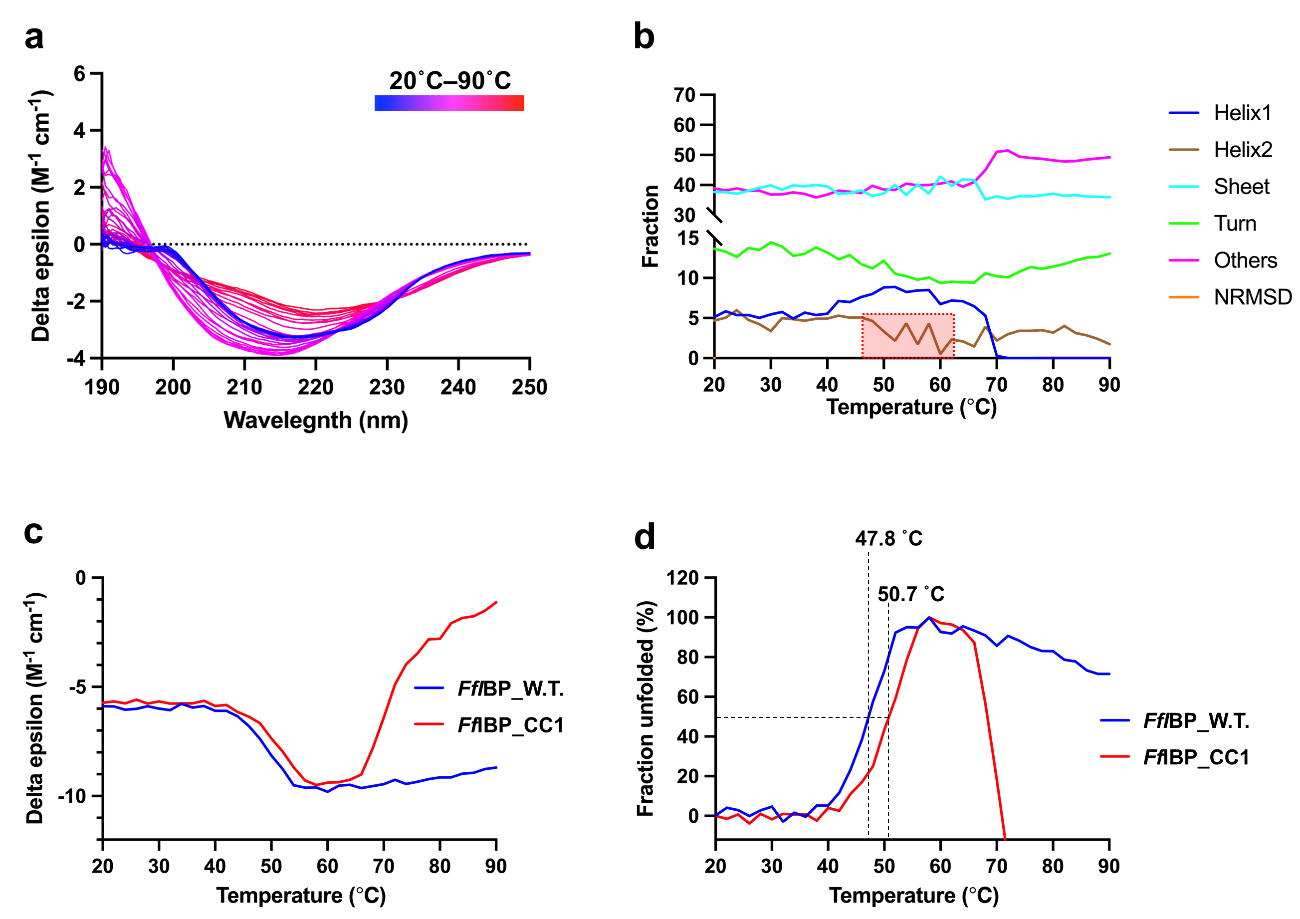


**Supplementary Figure S6.** Circular dichroism (CD) spectroscopy of *Ff*IBP_CC1 and comparison with *Ff*IBP during the unfolding process. (**a**) Temperature-dependent CD spectra of *Ff*IBP_CC1 from 20 to 90 °C. Spectra were recorded in 10 mM Tris-HCl (pH 8.5) buffer from 190 to 250 nm with 0.5 nm steps and 0.5 sec/step at a 1 mm path length. (**b**) Secondary structure contents as a function of temperature. Amino acid propensities for helix, β-strand, turn, and other (mostly coil) structures at different temperatures were calculated using BeStSel36 and indicated with different color codes. (**c**, **d**) CD spectra demonstrating the thermal denaturation of *Ff*IBP and *Ff*IBP_CC1. Changes in the secondary structure of proteins were monitored by following the changes in delta epsilon at 210 nm.


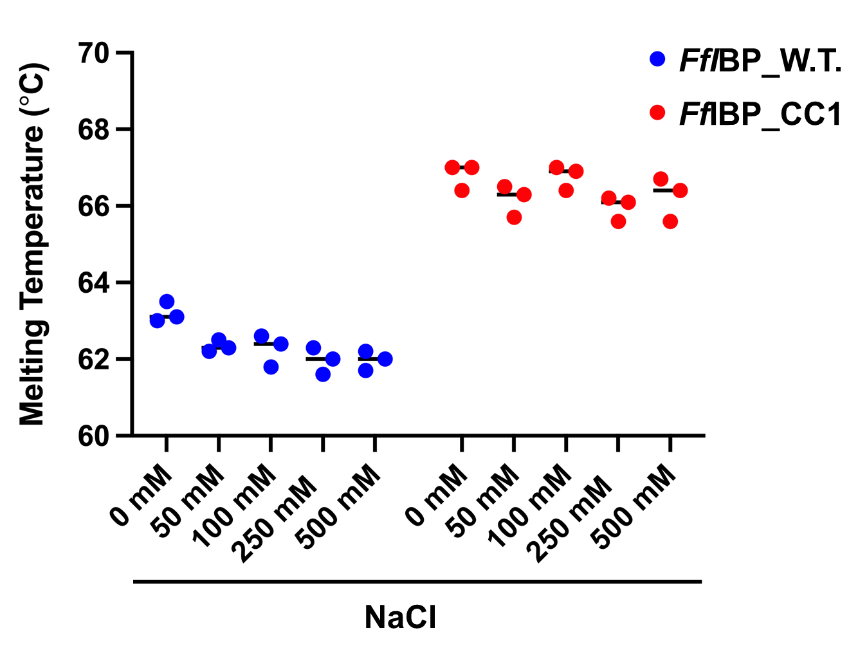


**Supplementary Figure S7.** Thermal stability assay at different salt (NaCl) concentrations using Tycho NT.6. Black colored line: average of triplicates.
